# Herbal formulas for detoxification and dredging collaterals in treating carotid atherosclerosis: a systematic review and meta-analysis

**DOI:** 10.3389/fphar.2023.1147964

**Published:** 2023-12-11

**Authors:** Leyi Zhang, Xing Huang, Yonghong Gao, Xiangyu Li, Qiao Kong, Ying Chen, Jingling Chang, Genming Zhang, Yan Ma

**Affiliations:** ^1^ Department of Neurology, Dongzhimen Hospital, Beijing University of Chinese Medicine, Beijing, China; ^2^ Department of Neurology, Beijing University of Chinese Medicine Third Affiliated Hospital, Beijing, China; ^3^ Key Laboratory of Chinese Internal Medicine of Ministry of Education Dongzhimen Hospital, Beijing University of Chinese Medicine, Beijing, China; ^4^ Department of Global Health, School of Public Health, Health Science Centre, Beijing, China; ^5^ Department of Pathophysiology and Allergy Research, Center for Pathophysiology, Infectiology and Immunology, Medical University of Vienna, Vienna, Austria

**Keywords:** carotid atherosclerosis, herbal formulas, detoxification and dredging collaterals, systematic review, meta-analysis

## Abstract

**Objective:** To systematically evaluate the efficacy and safety of the Chinese medicine detoxification and dredging collaterals in treating carotid atherosclerosis (CAS).

**Methods:** A systematic and comprehensive search of nine relevant domestic and international databases were conducted from their inception until June 2022. The methodological quality of the included trials was evaluated, and the efficacy and safety were comprehensively analyzed. After applying the inclusion and exclusion criteria to the randomized controlled trials (RCTs), the research quality evaluation and data extraction were conducted, followed by a meta-analysis of the selected articles. The Cochrane’s Bias risk assessment was utilized to evaluate the quality of the evidence.

**Results:** Of the 2,660 studies initially retrieved, 14 studies were included, involving a total of 1,518 patients. The results of the meta-analysis indicated that the clinical efficacy of the Detoxification and Collateral Dredging method in the treatment of CAS was superior to that of western medicine treatment alone, and the difference was statistically significant [RR = 1.23, 95% CI (1.13, 1.34)] Furthermore, carotid intima-media thickness [Mean Difference (MD) = −0.10, 95% CI (−0.13, −0.08)] and Crouse plaque score [MD = −0.54, 95% CI (−0.75, −0.32)] were significantly lower in the Detoxification and Collateral Dredging group compared to the pure western medicine treatment group. The difference was statistically significant. In addition, serum total cholesterol [MD = −0.70, 95% CI (−0.85, −0.55)] and low-density lipoprotein cholesterol [MD = −0.70, 95% CI (−0.85, −0.55)] were lower in the Detoxification and Collateral Dredging group than in the Western medicine group, with all differences being statistically significant. Serum high-density lipoprotein cholesterol was higher in the Detoxification and Collateral Dredging group compared to the pure western medicine group, and the difference was statistically significant [MD = 0.17, 95% CI (0.11, 0.23)].

**Conclusion:** The use of Chinese medicine Detoxification and Collateral Dredging approach in the treatment of CAS may offer benefits in improving carotid atherosclerotic plaque and reducing blood lipid levels, with a safety profile superior to that of western medicine treatment alone.

## 1 Introduction

Atherosclerosis (AS) is a chronic inflammatory disease primarily driven by lipid deposition, typically resulting in plaque formation within large and medium-sized arteries (Libby, 2021); it stands as a major cause of ischemic heart disease and stroke (Soehnlein and Libby, 2021). AS often manifests in coronary, brain, and carotid arteries, mainly due to lipid deposition and plaque formation. This occurs in regions characterized by relatively slow blood flow at the carotid bifurcation, rapid expansion of blood vessel diameter, and potential development of vortexes in blood flow (Chen and Hu, 2017). Carotid atherosclerosis (CAS) refers to a chronic inflammatory disease where the carotid arteries are affected by AS due to various factors, leading to arterial lumen obstruction. The global prevalence of CAS among people aged 30–90 years reached 27.6% in 2020, affecting more than one billion people worldwide (Song et al., 2020). Additionally, the pathogenesis and risk factors of CAS are complicated and challenging to control (Du et al., 2019). CAS significantly increases the risk of acute cerebral infarction (Clarke et al., 2017) and contributes to the morbidity and mortality of CAS-induced vascular diseases (Hou et al., 2018). Thus, CAS has become a health burden worldwide. Conventional treatments, such as lipid-regulating, antiplatelet, and antioxidant drugs, have some drawbacks and practical challenges, such as extended treatment cycle durations, high economic cost, variable efficacy, and poor patient compliance. Therefore, researchers seek a safer alternative treatment for CAS (Virani et al., 2021).

Chinese botanical drug rooted in traditional Chinese medicine (TCM) have become widely used in China and other Asian countries for the treatment of atherosclerotic diseases (Marshall, 2020). In Asia, TCM has been actively used in the treatment of CAS and has demonstrated significant therapeutic efficacy with minimal side effects (Sun et al., 2021), especially in the application of Chinese botanical drug characterized by the therapeutic principle of detoxifying and dredging collaterals (Wang et al., 2018). Several experts have proposed the pathogenesis hypothesis, suggesting that toxins damage brain collaterals. The toxic and pathological products can lead to improper functioning of the Zang-fu organs, qi, and blood, which can destroy the body and damage the brain collaterals (Wang Y., 1997). Presently, toxin is an important pathological factor that is involved throughout the occurrence and development of CAS and its pathogenesis. Stasis is an important stage in CAS disease pathogenesis, whereas toxic damage and collateral resistance are majorly involved in atherosclerosis pathogenesis. Consequently, clinical practitioners frequently employ detoxifying and collateral dredging TCM as therapeutic strategies for managing this disease.

With ongoing advancements in research elucidating the mechanisms of toxin-induced cerebral collateral damage and the theory surrounding toxin elimination and collateral clearance, a substantial body of both fundamental and clinical research has confirmed the association between toxin-induced damage to brain collaterals and CAS pathogenesis. Furthermore, these studies have underscored the significance of toxin removal and collateral clearance in preventing CAS-related disorders and elucidated the corresponding therapeutic mechanisms (Zhou et al., 2017). At present, the commonly used traditional Chinese medicine can be divided into activating blood circulation and removing blood stasis, activating blood circulation and removing blood circulation and removing phlegm, clearing heat, tonifying deficiency, resolving phlegm, promoting qi, warming tong, clearing heat, etc., The main treatment is removing phlegm, stasis, heat, turbidizing fire and other toxic pathogens. Thus, there is a substantial practical need for application of Chinese herbal formulas with detoxifying and dredging collateral properties for early intervention of CAS; this may hold the potential to advance the timing of cerebrovascular disease intervention and prevent the onset of cerebrovascular diseases. Although the method of detoxifying and dredging collaterals in preventing CAS has been partially validated for its preventive role in CAS, its potential additional effects have not been systematically evaluated. Therefore, this systematic review aimed to summarize the evidence on the detoxifying and collateral dredging therapy in the treatment of CAS, providing both patients and healthcare professionals with enhanced understanding and guidance for informed clinical decision-making.

## 2 Methods

### 2.1 Search strategy

Various literature on clinical randomized controlled trials published in public databases and journals that used detoxification and dredging collateral therapy for CAS were searched. Chinese databases included the China National Academic Library (CNKI), Wanfang Database, VIP Chinese Science and Technology Journal Database, and China Biomedical Literature Database, whereas the English databases included the Web of Science database, PubMed, Medline, and Embase. The search period was from the time of establishment of each library to 1 June 2022.

### 2.2 Literature search scope and strategy

For the Chinese database, we included “detoxification,” “dredging collaterals,” “TCM and arterial stenosis,” “arterial plaque,” “atherosclerosis,” “lipid metabolism disorder,” and “neck” as search terms. For the English database, we included “Chinese traditional,” “Drugs,” “Chinese herbal,” “Detoxification,” “dredging collaterals,” “TCM and arterial stenosis,” “arterial plaque,” “atherosclerosis,” “lipid metabolism disorder and “neck,” and “CASs” as search terms. The detailed search strategy is shown in the Supplementary Appendix. There was no restriction on the type and language of the publications.

### 2.3 Inclusion and exclusion criteria

#### 2.3.1 Inclusion criteria

Type of study: Randomized controlled trials (RCTs), which were generated in a random sequence with explicit reference to randomization methods, both blinded and not blinded; 2) Study participants: Western medicine diagnosis, including CAS diagnosis carotid intima-media thickness (IMT) > 1.0 mm according to the Chinese expert’s recommendations for the diagnosis and treatment of CAS diseases in the elderly (Wang Y., 1997); 3) Plaque: a) Localized IMT thickening protruding into the arterial lumen >0.5 mm, b) IMT increased by >50% compared to the surrounding area, and c) IMT >1.5 mm. Patients who met one of the above three conditions were included; 4) Intervention measures: a) The experimental group was treated with TCM detoxification and dredging collaterals alone (the methods of clearing heat and detoxification, promoting blood circulation and removing blood stasis, searching for wind and resolving spasm, and removing blood stasis and dredging collaterals advocated in the theory of toxin damage of brain collaterals in the treatment of cerebrovascular disease), or in combination with conventional western medicine treatment, regardless of the mode of administration, dosage form, and composition of TCM; b) The control group was treated with placebo or conventional western medicine treatment, without any TCM ingredients or their extracts. Western medicine routine treatment included antiplatelets, β-receptor blockers, statins, and other commonly used drugs; 5) Outcomes: a) Primary outcome: common carotid artery IMT, blood lipid indicators [low-density lipoprotein cholesterol (LDL-C), high-density lipoprotein cholesterol (HDL-C), triglyceride (TG), and total cholesterol (TC)]; b) Secondary outcomes included total PA (mm^2^), number of plaques (pieces), Crouse plaque score, effective rate, and adverse reactions. All primary outcome measures were reported for the outcome measures of the included studies.

#### 2.3.2 Exclusion criteria

1) Repeated publications, 2) studies where the original literature data is incomplete or the full text cannot be obtained completely, and the baselines are not comparable, and 3) the main research indicators or data are unavailable in the research protocol.

### 2.4 Literature screening and data extraction

The literature screening method was as follows: Two researchers independently screened the literature in the database, created a new database using the Note Express software to manage the literature, and screened the research according to the inclusion and exclusion criteria. The titles and abstracts were carefully read to exclude ineligible literature and classify the research types. The full texts of the initially screened literature were also read while data were extracted from the included literature before recording the unincorporated literature and the reasons in detail.

Data extraction was completed independently by two reviewers (Xiangyu Li and Qiao Kong) to ensure accuracy and completeness of the extracted data. A literature feature database was created using Microsoft Office Excel 2019, and the title, author, and year of each study were consecutively recorded. Age, number of participants, dropout, intervention measures, treatment course of observation, and total sample size were recorded separately. The diagnostic criteria and observation indexes of western medicine and TCM used in the literature were recorded, as well as the IMT, blood lipid indexes (LDL- C, HDL-C, TG, and TC), total PA (mm^2^), plaque number (pieces), Crouse score, effective rate, and adverse reactions. The composition of TCM prescriptions was also recorded. Methodological randomization, blinding, allocation concealment, data integrity of results, selective reporting of results, and other sources of bias were well documented.

### 2.5 Evaluation of literature quality

For literature analysis, the Review Manager 5.4 (RevMan 5.4) software provided on the Cochrane website was referenced. The included studies were assessed for risk of bias, which was examined according to the intervention reviews in the Cochrane Systematic Reviewer’s Handbook (Cochrane, 2023). There were seven items in total: random allocation method, allocation scheme concealment, subjects’ blinding, investigator blinding, data integrity of results, selective reporting of study results, and other biases (such as inadequate information, claims of deception, and potential sources of bias related to the specific study design used among others). Two postgraduate students independently assessed the bias of the selected studies according to the contents suggested in the manual. In case of disagreement, the supervisor discussed the study after synthesizing the opinions of both parties and provided the final degree of bias risk. Finally, the judgment of “low risk of bias,” “high risk of bias,” and “uncertain risk of bias” was made for the literature. We followed the Preferred Reporting Items for Systematic Review and Meta-Analysis (PRISMA) checklist to complete the meta-analysis. The reporting of this systematic review was guided by the PRISMA guidelines.

### 2.6 Statistical analysis

The RevMan5.4 software was used for statistical analysis, and the included studies were first evaluated for heterogeneity. If *I* ^*2*^ < 50%, the study was considered to have no significant heterogeneity, and if *I* ^*2*^ > 50%, the study was considered to have significant heterogeneity. If the outcome is a dichotomous variable, the heterogeneity test was performed first. *p* > 0.1 and *I* ^*2*^ < 50% indicate acceptable heterogeneity, and a fixed effects model was used for meta-analysis. For *p* ≤ 0.1 or *I* ^*2*^ ≥ 50%, an overall analysis was conducted, and the source of heterogeneity was explored. If the source of heterogeneity can be found, the study was checked if it should be divided into analyses according to the source of heterogeneity. For continuous numerical variables, if the observation index was a continuous variable when the baseline table scores of the experimental and control groups included in the literature were uneven, the baseline change value was considered for the analysis, and ratings were compared. The relative risk (*RR)* was selected as the effect index for the enumerated data, and the weighted mean difference (*WMD)* was selected as the effect index for the measured data. The 95% confidence interval (*CI*) was calculated for both, and the difference was considered statistically significant if *p* < 0.05.

## 3 Results

### 3.1 Literature search results

**FIGURE 1:**
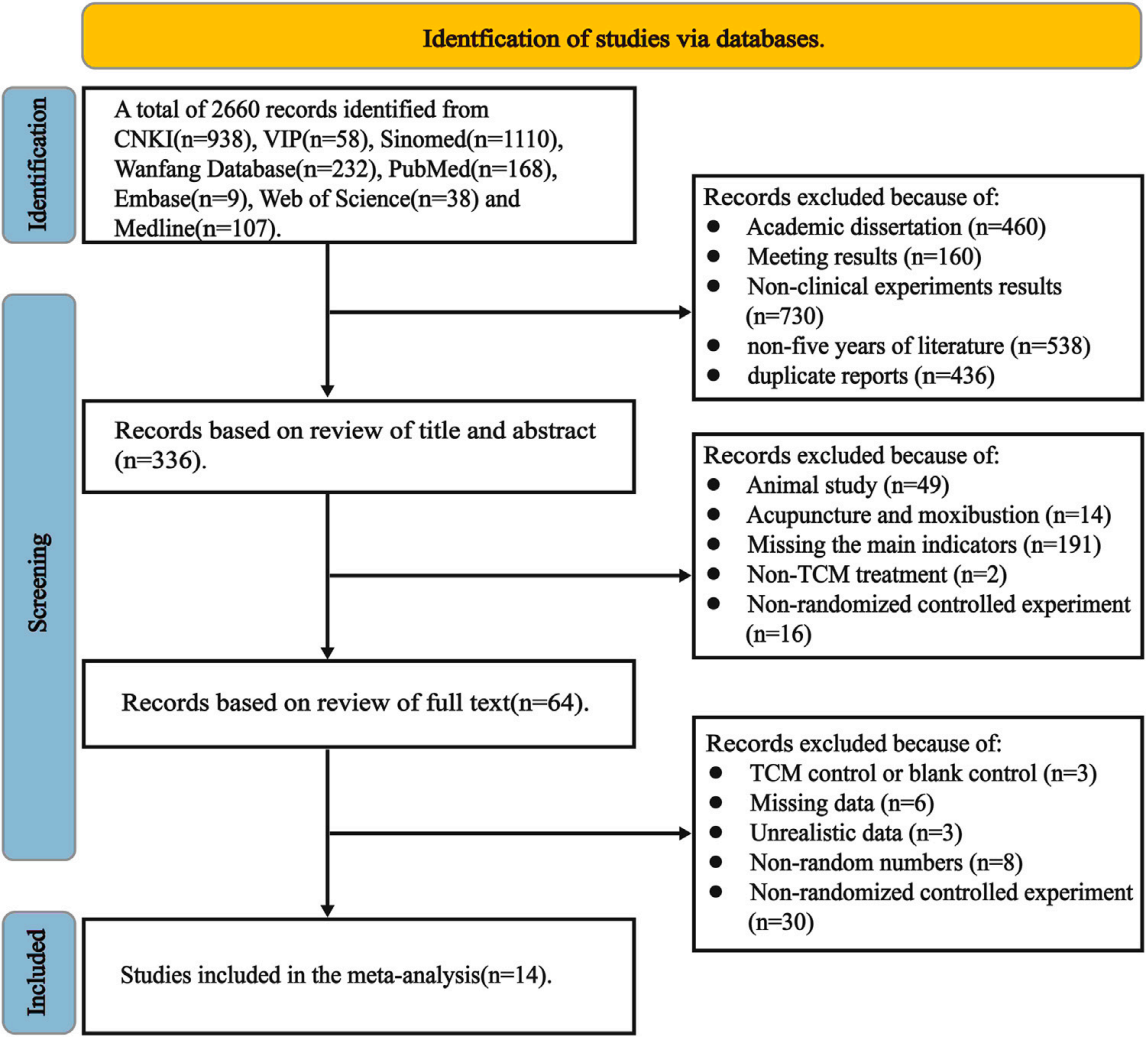
Trial flow diagram.

A total of 2,660 documents were retrieved: 460 dissertations, 160 conference results, 730 non-clinical experiments, 538 duplicate documents, and 436 non-recent five-year documents. Of these, 336 were initially obtained. A total of 322 articles were excluded: 49 articles on animal experiments, 14 articles on acupuncture and moxibustion, 191 articles on inconsistent diagnosis, 16 articles on non-RCTs, two articles on non-TCM treatment, three articles on TCM control or blank control, six articles with missing indicators, three unknown data articles, 8 with non-random numbers, 30 non-RCTs, and 14 included literature (Figure 1).

### 3.2 Features of the included literature

Fourteen papers included in this study were completed between 2017 and 2022, with sample sizes of 36–268 cases. The experimental groups included in the literature were treated with a Chinese herbal formula for detoxification and dredging collateral or a Chinese herbal formula combined with western medicine intervention, while the control groups were treated with western medicine and placebo. All 14 selected studies were in line with the Chinese expert suggestions on the diagnosis and treatment of CAS diseases in the elderly. The diagnostic criteria were middle CAS, that is, patients with carotid IMT >1.0 mm, which can be diagnosed as carotid intima-media thickening and is consistent with the localized IMT protruding into the arterial lumen greater than carotid intima-media plaques. The latter can be diagnosed in patients with one of three parameters: 0.5 mm, IMT increased by > 50% compared with the surrounding area, and IMT >1.5 mm.

Of these studies, 10 adopted the TCM diagnostic criteria, 1 article adopted the syndrome section of TCM clinical diagnosis and treatment terms, one article adopted the TCM syndrome differential diagnosis, and one adopted atherosclerosis-integrated TCM and western medicine diagnosis and treatment based on expert consensus. Six studies adopted guidelines for clinical research of new Chinese herbal formulas, and one adopted guidelines for clinical research of new Chinese herbal formulas in treating chest arthritis (angina pectoris of coronary heart disease) and TCM standards for coronary heart diseases.

A total of 1,596 participants were included, of which 25 dropped out (15 and 10 from the observation and control groups, respectively). Finally, 1,571 valid samples were included, of which 785 and 786 patients were included in the experimental and 786 control groups, respectively. The characteristics of the participants included in the study are shown in Table 1, and the outcomes and diagnostic criteria are presented in Table 2. Herbal formulas for Detoxification and Dredging collaterals of each original studies were reported in detail in Supplementary Appendix S1.

**TABLE 1:** Characteristics of study participants.

	Experimental group						Control group				
Author + year	Age (year)	Sex (Male/Female)	Drop	Intervention	Course (months)		Age (year)	Sex (Male/Female)	Drop	Intervention	Course (months)
Su et al. (2021)	65.56 ± 6.79	7/11	0	TCM	12		65.56 ± 6.79	7/11	0	Placebo	12
Cheng (2017)	61.30 ± 11.30	56/46	0	TCM + WM	24		62.4 ± 12.6	57/45	0	WM	24
Xie and Guo (2018)	58.06 ± 8.82	16/19	1	TCM + WM	12		60.03 ± 9.37	19/15	2	WM	12
(Chen et al., 2017)^]^	64.50 ± 12.80	63/69	8	TCM + WM	12		63.8 ± 13.6	64/71	3	WM	12
Cao (2021)	64.75 ± 3.19	41/51	0	TCM + WM	24		62.19 ± 2.81	37/49	0	WM	24
Liu et al. (2021a)	61.70 ± 5.90	28/13	0	TCM + WM	8		61.5 ± 5.8	28/12	0	WM	8
Kand et al. (2020)	58.35 ± 9.93	23/17	0	TCM + WM	24		57.58 ± 9.86	22/18	0	WM	24
Zhang et al. (2020)	49.74 ± 6.81	17/13	0	TCM	2		49.16 ± 6.37	18/12	0	WM	2
Xin et al. (2020)	56.85 ± 4.13	27/19	0	TCM + WM	12		57.31 ± 4.20	25/21	0	WM	12
Bai and Luo (2019)	52.10 ± 5.30	10/12	0	TCM	20		52.8 ± 6.1	12/10	0	Placebo	20
Shi and Chang (2019)	61.27 ± 2.67	29/26	0	TCM + WM	12		61.03 ± 2.33	30/25	0	WM	12
Cao et al. (2019)	56.40 ± 3.60	45/33	0	TCM + WM	2		56.2 ± 3.3	47/31	0	WM	2
Wang (2020)	66.20 ± 5.00	24/16	0	TCM + WM	12		66.4 ± 5.1	25/15	0	WM	12
Liu et al. (2019)	61.28 ± 8.69	42/26	6	TCM + WM	16		60.53 ± 9.28	41/25	5	WM	16

*Note:* TCM: traditional chinese medicine; WM: western medicine.

**TABLE 2:** Outcome indicators and diagnostic criteria used in this study.

Author + year	Main outcome indicators	Secondary outcome indicators
Su et al. (2021)	IMT, TC, TG, LDL-C, HDL-C	Crouse plaque score, Effective rate, PA, Adverse drug reactions
Cheng (2017)	IMT, TC, TG, LDL-C, HDL-C	Crouse plaque score, Effective rate
Xie and Guo (2018)	IMT, TC, TG, LDL-C, HDL-C	Crouse plaque score, Adverse drug reactions
(Chen et al., 2017)	IMT, TC, TG, LDL-C	Number of plaque, Effective rate, Adverse drug reactions
Cao (2021)	IMT, TC, TG, LDL-C, HDL-C	PA
Liu et al. (2021a)	IMT, TC, TG, LDL-C, HDL-C	PA, Number of plaque, Effective rate, Adverse drug reactions
Kand et al. (2020)	IMT, TC, TG, LDL-C	Crouse plaque score, Effective rate
Zhang et al. (2020)	IMT, TC, TG, LDL-C	Effective rate, Adverse drug reactions
Xin et al. (2020)	IMT, TC, TG, LDL-C, HDL-C	PA, Adverse drug reactions
Bai and Luo (2019)	IMT, TC, TG, LDL-C	--
Shi and Chang (2019)	IMT, TC, TG, LDL-C, HDL-C	Crouse plaque score
Cao et al. (2019)	IMT, TC, TG, LDL-C, HDL-C	Effective rate, Adverse drug reactions
Wang (2020)	IMT, TC, LDL-C, HDL-C	Effective rate, PA
Liu et al. (2019)	IMT, TC, TG, LDL-C, HDL-C	Crouse plaque score, Number of plaque

***Note:*** IMT: Intima-media thickness, TC: serum total cholesterol, TG: triglycerides, LDL-C: Low-density lipoprotein cholesterol, HDL-C: High-density lipoprotein cholesterol.

### 3.3 Methodological quality evaluation of the included literature

**FIGURE 2:**
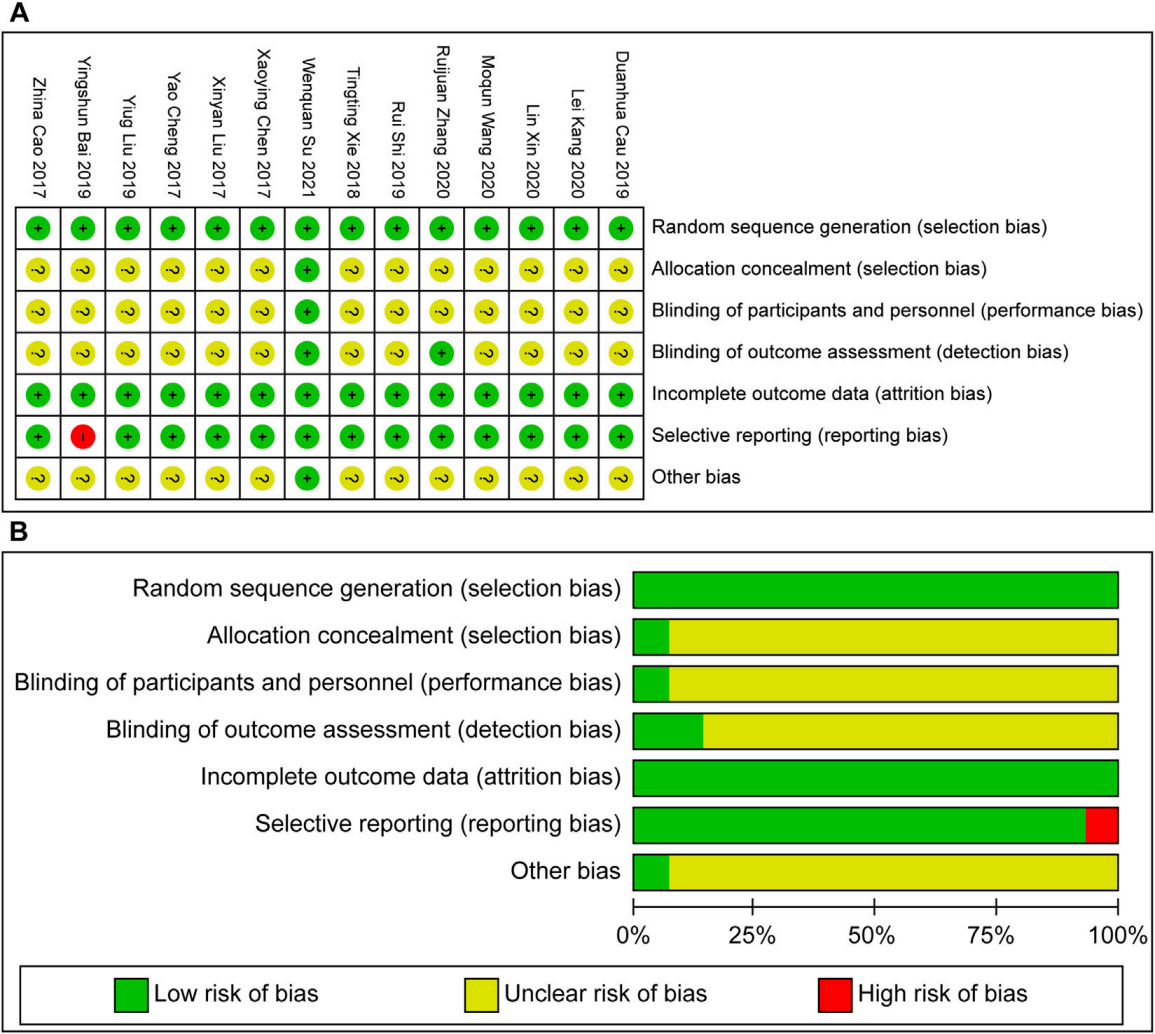
**(A)** Bias risk table risk. **(B)** Assessment result map.

Risk assessment was performed according to Cochrane’s bias risk assessment tool in the review manager, and the results were imputed to generate a bias risk table (Figure 2A) and a risk assessment result map (Figure 2B). Owing to the peculiarity of the TCM decoction intervention, ignoring the subjects of the TCM decoction in the randomized controlled study was difficult. Therefore, according to the contents of the research, the operator was not ignored during the implementation of the trial. The basis of the risk of bias is presented in Table 3.

**TABLE 3:** Basis for risk of bias.

Author + year	Random sequence generation	Blinding	Allocation concealment	Incomplete outcome data assessments	Selective reporting
Su et al. (2021)	Randomized block	Double Blind	Random coding table	Yes	No
Cheng (2017)	Random number table	NR	NR	Yes	No
Xie and Guo (2018)	Random number table	NR	NR	Yes	No
(Chen et al., 2017)	Random number table	NR	NR	Yes	No
Cao (2021)	Random number table	NR	NR	Yes	No
Liu et al. (2021a)	Random number table	NR	NR	Yes	No
Kand et al. (2020)	Random number table	NR	NR	Yes	No
Zhang et al. (2020)	Random number table	NR	NR	Yes	No
Xin et al. (2020)	Random number table	NR	NR	Yes	No
Bai and Luo (2019)	Randomized block	NR	NR	Yes	Yes
Shi and Chang (2019)	Random number table	NR	NR	Yes	No
Cao et al. (2019)	Random number table	NR	NR	Yes	No
Wang (2020)	Random number table	NR	NR	Yes	No
Liu et al. (2019)	Random number table	NR	NR	Yes	No

### 3.4 Effects of interventions

#### 3.4.1 IMT

**FIGURE 3:**
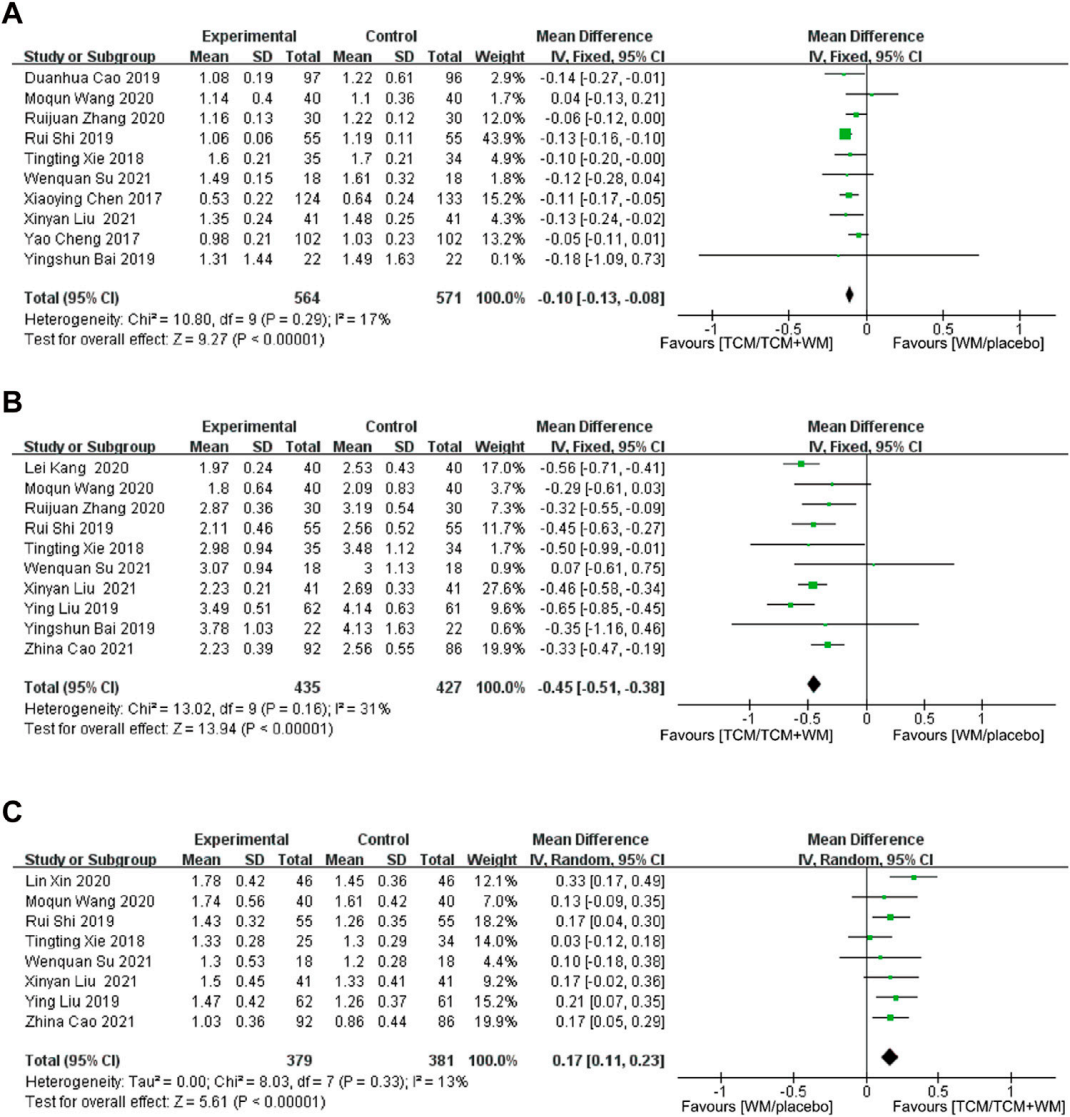
**(A)** Forest plot for IMT; **(B)** forest plot for LDL-C; **(C)** forest plot for HDL-C.

All 14 trials were included in this analysis. Significant heterogeneity was observed among the studies (*p* < 0.00001, *I* ^*2*^ = 82%), with a statistically significant difference [*p* < 0.00001, *MD* = −0.15, 95% *CI* (−0.20, −0.10)]. The RevMan software was used for the sensitivity analysis of the studies. After excluding the four studies with high heterogeneity, the results showed a reduced heterogeneity (*p* = 0.29, *I* ^*2*^ = 17%). Considering the heterogeneity source, the treatment time of Cao, (2021), Kand et al. (2020) was 6 months; for Liu et al. (2019), the time was 16 weeks, and Xin et al. (2020) was treated for CAS with high blood pressure. A total of 10 studies were included for analysis, of which 564 cases were in the observation group and 571 cases in the control group. According to *p* = 0.29, *I* ^*2*^ = 17%, the fixed effects model was selected for analysis. The results showed that the experimental group had a significant advantage in reducing IMT value compared with the control group (*p* < 0.00001, *MD* = −0.10, 95% *CI* [−0.13, −0.08]) (Figure 3A).

#### 3.4.2 LDL-C

All 14 trials were included in this analysis. Significant heterogeneity was observed among the studies (*p* < 0.00001, *I* ^*2*^ = 91%), with a statistically significant difference [*p* < 0.00001, *MD* = −0.50, 95% *CI* (−0.69, −0.32)]. Sensitivity analysis was performed. After excluding four studies that had high quality, the results showed reduced heterogeneity (*p* = 0.16, *I* ^*2*^ = 31%). Considering the heterogeneity source, the 2-week treatment time of Cao Duanhua was notable; for Cheng Yao, the treatment time was 24 weeks. In the study by Xin et al. (2020) and Chen et al. (2017) were treated for hypertensive CAS and type 2 diabetes with CAS, respectively. Finally, 10 studies were included for analysis. A total of 435 and 427 participants were used in the experimental and control groups, respectively. The fixed effects model was used. The results showed that the experimental group could effectively reduce the LDL-C level and was significantly better than the control group [*p* < 0.00001, *MD* = −0.45, 95% *CI* (−0.51, −0.38)] (Figure 3B).

#### 3.4.3 HDL-C

A total of 10 studies reported HDL-C. Meta-analysis showed a significant heterogeneity among them (*p* < 0.00001, *I* ^*2*^ = 95%), with statistically significant results [*p* = 0.01, *MD* = 0.28, 95% *CI* (0.07, 0.49)]. Sensitivity analysis showed that after excluding the two studies with high heterogeneity, the overall value was significantly reduced (*p* = 0.33, *I* ^*2*^ = 13%). Considering the heterogeneity source, the 2-week treatment time of Cao et al. (2019) was again influential, and that of Cheng, (2017), which was for 24 weeks. Eight studies were included, with 379 and 381 participants in the experimental and control groups, respectively. The fixed effects model was used. The results showed that the HDL-C in the experimental group was significantly higher than that in the control group [*p* < 0.00001, *MD* = 0.17, 95% *CI* (0.11, 0.23)] (Figure 3C).

#### 3.4.4 TC

**FIGURE 4:**
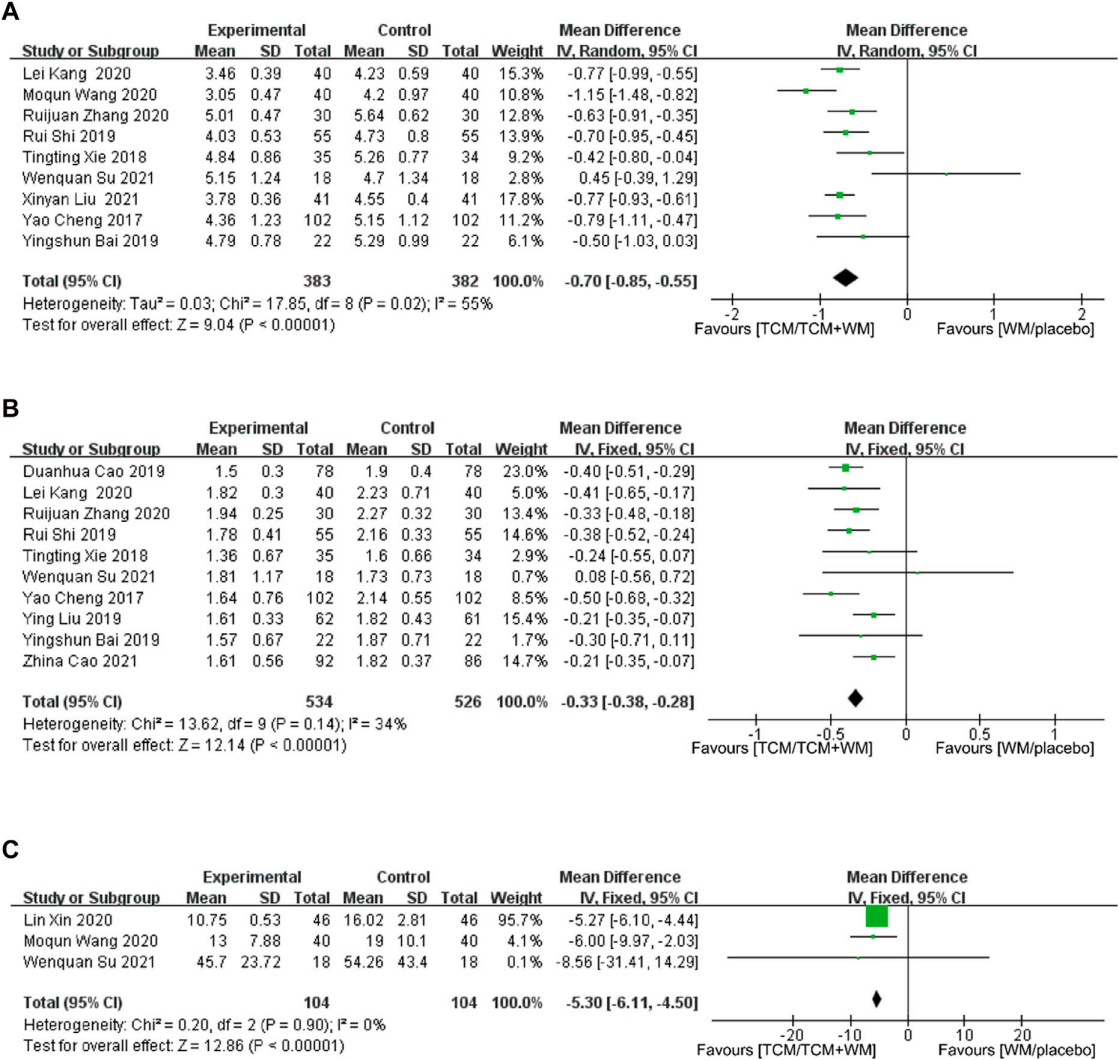
**(A)** Forest plot for TC; **(B)** forest plot for TG; **(C)** forest plot for plaque area.

A total of 14 studies reported TC. The results showed significant heterogeneity among the studies (*p* < 0.00001, *I* ^*2*^ = 97%). The random effect model was used for analysis, indicating a statistically significant difference [*p* < 0.00001, *MD* = −0.77, 95% *CI* (−1.11, −0.42)]. To explore the causes of heterogeneity, sensitivity analysis was performed, resulting in reduced heterogeneity (*p* = 0.02, *I* ^*2*^ = 55%), while the five studies with high heterogeneity were excluded. Considering the sources, the treatment times in the study by Cao et al. (2019), Liu et al. (2019), Cao (2021), were 2, 16 weeks, and 6 months, respectively. Kand et al. (2020) and Chen et al. (2017) treated for hypertensive CAS and type 2 diabetes with CAS, respectively. Nine studies were included, with 383 and 382 participants in the experimental and control groups, respectively. The random effect model was used, with the results showing that the experimental group had more advantages in reducing TC level than the control group [*p* < 0.000001, *MD* = −0.70, 95% *CI* (−0.85, −0.55)] (Figure 4A).

#### 3.4.5 TG

A total of 13 studies reported TG levels. Meta-analysis showed a large heterogeneity among the studies (*p* < 0.00001, *I* ^*2*^ = 95%). The random effect model was used for analysis, indicating a statistically significant difference [*p* < 0.00001, *MD* = −0.40, 95% *CI* (−0.60, −0.20)]. Sensitivity analysis showed that after excluding the three studies with high heterogeneity, the overall heterogeneity level was reduced significantly (*p* = 0.14, *I* ^*2*^ = 34%). Considering the heterogeneity source, Xin et al. (2020) and Cheng, (2017) treated for hypertension CAS and type 2 diabetes with CAS, respectively, and the source of heterogeneity was not found in the study of Liu Xinyan (Liu X. et al., 2021). The 10 studies had a total of 534 and 526 participants in the experimental and control groups, respectively. The fixed effects model was used for analysis. The results showed that the effect of the experimental group in reducing TG level was more obvious than that of the control group [*p* < 0.000001, *MD* = −0.33, 95% *CI* (−0.38, −0.28)] (Figure 4B).

#### 3.4.6 Plaque area

Five studies reported atherosclerotic PA. The meta-analysis results showed great heterogeneity among the studies (*p* < 0.001, *I* ^*2*^ = 98%), and the random effect model was used for analysis. The difference was statistically significant [*p* = 0.01, *MD* = −3.12, 95% *CI* (−5.54, −0.71)]. According to sensitivity analysis, the treatment time was considered the source of heterogeneity. The duration of the treatment were 8 weeks and 6 6 months in a study by Liu X. et al. (2021) and Cao, (2021), respectively, and the rest were 12 weeks. Two studies with high heterogeneity were excluded, leaving three studies included for analysis. The results showed no heterogeneity among the studies (*p* = 0.90, *I* ^*2*^ = 0%). The fixed effects model was used for analysis. The experimental group had a significant advantage in reducing the plaque area compared with the control group [*p* < 0.00001, *MD* = −5.30, 95% *CI* (−6.11, −4.50)] (Figure 4C).

#### 3.4.7 The number of plaques

**FIGURE 5:**
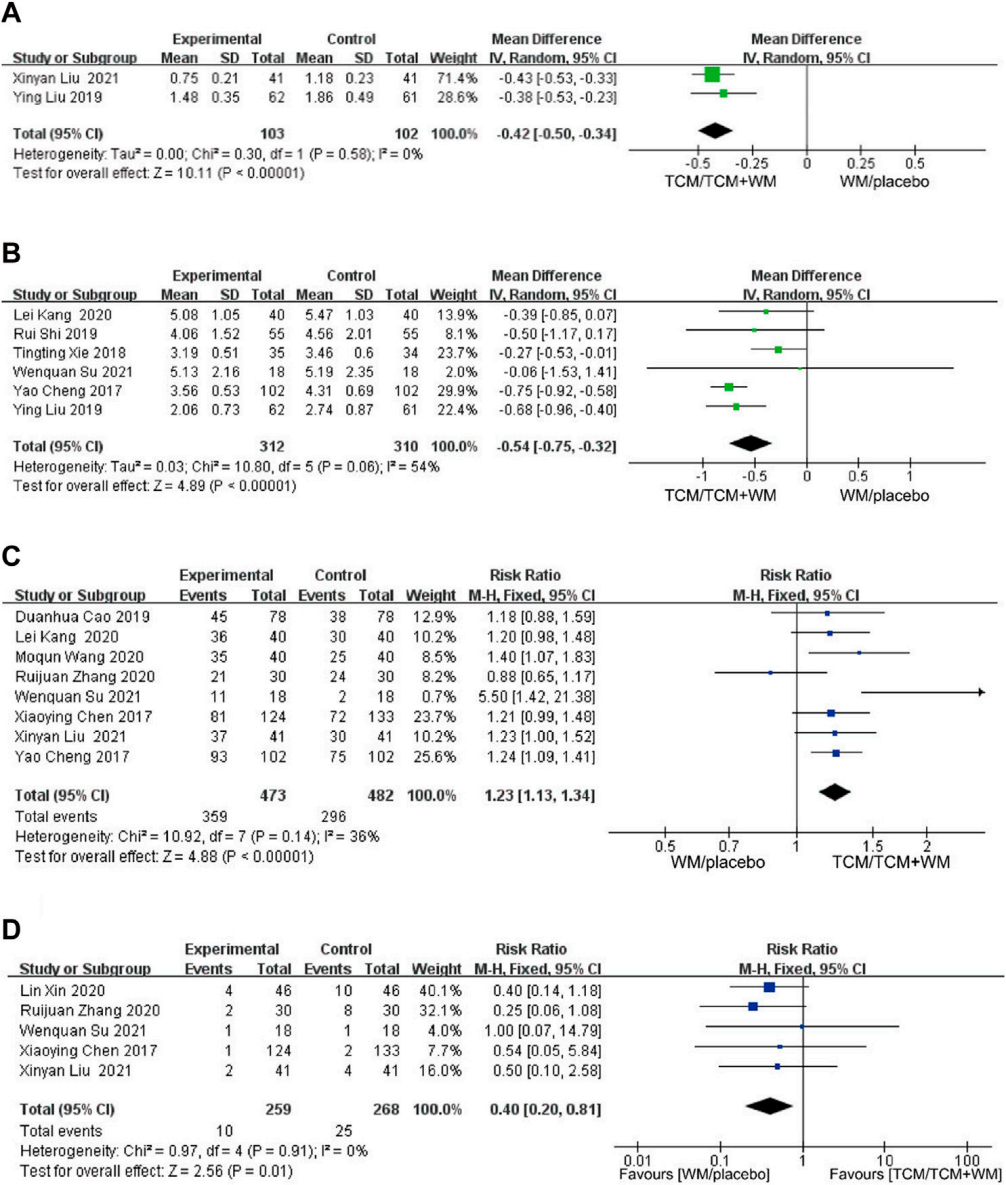
**(A)** Forest plot for number of plaque; **(B)** forest plot for crouse plaque Score; **(C)** forest plot for efficiency. **(D)** forest plot for adverse reactions.

Three studies reported a number of plaques. The results showed strong heterogeneity among them (*p* < 0.00001, *I* ^*2*^ = 91%). The random effect model was used for analysis, indicating a statistically significant difference [*p* = 0.04, *MD* = −0.45, 95% *CI* (−0.86, −0.03)]. A sensitivity analysis was conducted. After one study was excluded, the heterogeneity disappeared (*p* = 0.58, *I* ^*2*^ = 0%). Considering that the source of heterogeneity was the disease treatment, Cheng, (2017) was treated for type 2 diabetes with CAS. The fixed effects model was used for analysis. The results showed that the difference had a statistical effect [*p* < 0.00001, *MD* = −0.42, 95% *CI* (−0.50, −0.34)], indicating that TCM combined with western medicine could reduce the number of plaques, compared to western medicine (Figure 5A).

#### 3.4.8 Crouse plaque score

Six studies reported the Crouse score. Meta-analysis showed that a certain heterogeneity among them (*p* = 0.06, *I* ^*2*^ = 54%). The random effect model was used for analysis, with a statistically significant difference between TCM and western medicine in reducing the Crouse score [*p* < 0.00001, *MD* = −0.54, 95% *CI* (−0.75, −0.32)] (Figure 5B).

#### 3.4.9 Efficiency and adverse reactions

Eight of the included studies reported an effective rate, which was a binary variable. Therefore, the RR value was used for the effect quantity without any significant heterogeneity among the studies (*p* = 0.14, *I* ^*2*^ = 36%). The fixed effects model was used for analysis, and the results were statistically significant [*p* < 0.00001, *RR* = 1.23, 95% *CI* (1.13–1.34)]. The results showed that the effective rate of the experimental group greatly improved compared with that of the control group (Figure 5C).

A total of five studies reported adverse reactions. One research (Su et al., 2021) reported a case of mild diarrhea in each group during the treatment. One study (Chen et al., 2017) reported a case of abdominal distension and nausea in the experimental group and two cases of abnormal liver function in the control group. One study reported that a case of constipation and one case of fatigue occurred in the experimental group (Liu X. et al., 2021), and two cases of constipation, one case of fatigue, and one case of abdominal pain occurred in the control group. One study (Zhang et al., 2020) reported one case each of nausea and vomiting and abdominal liver function in the experimental group and eight cases in the control group (nausea and vomiting, three; rash, one; abnormal liver function, four). One study (Xin et al., 2020) reported four cases in the experimental group (mild headache, two; insomnia, one; dry mouth, one) and 10 cases in the control group (headache, four; insomnia, three; dry mouth, two; fatigue, one). The most adverse reactions were gastrointestinal reactions, headache, and insomnia. No heterogeneity was observed between the two groups (*p* = 0.91, *I* ^*2*^ = 0%). With the fixed effects model, a statistically significant difference was observed in adverse reactions between the two groups, suggesting that TCM can reduce adverse reactions experienced with western medicine [*p* = 0.01, *RR* = 0.40, 95% *CI* (0.20–1.81)] (Figure 5D).

### 3.5 Publication bias

**FIGURE 6:**
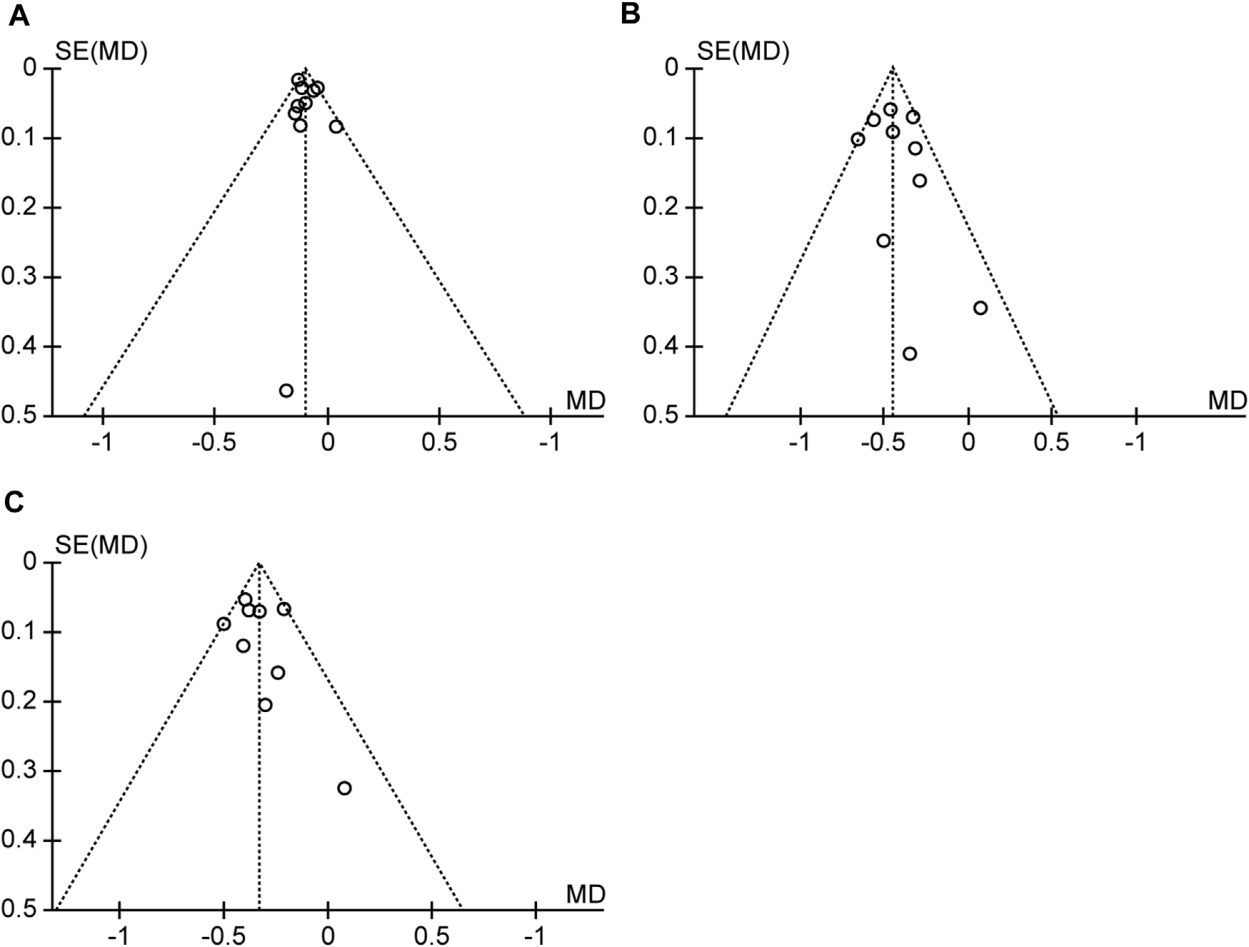
**(A)** Funnel plot for IMT. **(B)** Funnel plot for LDL-c. **(C)** Funnel plot for TG.

Funnel plot analysis was performed on the outcome indicators of IMT/LDL/TG studies included in the system evaluation (Figure 6). The left-right symmetry of the three funnel charts was poor, suggesting a certain degree of publication bias.

## 4 Discussion

### 4.1 Efficacy and safety of TCM detoxification and dredging collaterals on CAS

In this systematic review, we evaluated 14 studies on the effectiveness of detoxification and dredging collaterals used in TCM for the treatment of CAS. The traditional Chinese medicine used in the experimental group was mainly used to invigorate Qi and expel phlegm, invigorate Qi and activate blood circulation, and clear away heat and toxin. Its curative effect on carotid atherosclerotic plaque was better than that of the control group. It shows that traditional Chinese medicine can effectively reduce the area of carotid atherosclerotic plaque. The traditional Chinese medicine of detoxification and dredging collaterals is better than the control group in reducing blood lipid, which shows that the traditional Chinese medicine compound treatment is better than the simple western medicine group in regulating blood lipid. TCM, either independently or in combination with western medicine, guided by the principles of detoxification and collateral dredging, demonstrated a reduction in carotid IMT and favorable outcomes in related blood and scoring indicators. These results support the effectiveness of TCM, guided by the principles of detoxification and collateral dredging, in reducing CAS plaque. Therefore, a combined therapeutic approach of detoxification and collateral dredging from TCM and western medicine appears to be an appealing option for the management of CAS.

Regarding safety, five studies reported primarily mild adverse events, such as gastrointestinal reactions, headaches, and insomnia. However, in some studies where detoxification and collateral dredging herbal formulas were used in conjunction with western medicine, establishing a definitive causal relationship between these formulas and adverse events can be challenging. Additionally, some studies did not provide safety results for these therapeutics. Therefore, the safety results obtained from these studies need to be confirmed by future specialized safety evaluation studies.

14 articles are from China, so the prescriptions of Chinese medicines are mainly based on the relevant regulations of the Chinese Pharmacopoeia (2015). Although the original literature of 14 articles did not qualify and quantify the ingredients, each Chinese medicine in the prescriptions is in accordance with the Chinese Pharmacopoeia, and have a long history of extensive clinical experience and credible experimental results. Therefore, the Chinese Pharmacopoeia (Supplementary Appendix S01) were used for the standardization of the traditional chinese medicine name (Leong et al., 2020; Xu et al., 2021). A chemical profile following the standards established in the ConPhyMP (Heinrich et al., 2022) statement was detailed description in checkliset (Supplementary Appendix S02).

The pathogenesis of CAS in traditional Chinese medicine is “toxin damage and collateral obstruction,” which put forward a new apoplexy etiology theory, apoplexy toxicity heresy by Academician Wang Yongyan, a modern scholar (Wang YY., 1997), and its pathogenic factors mainly include phlegm toxicity, fire toxicity and blood stasis toxicity, they are important pathological products that destroy the body and damage the brain (Qu and Zhou, 2012).

These factors are caused by abnormal changes in the function of the body, resulting in undesirable products that can block blood vessels or affect vascular function. The theory of “toxic damage and collaterals obstruction” in traditional Chinese medicine is highly correlated with the mechanism of CAS: Toxic damage is the prerequisite to promote the pathological changes of CAS, which is consistent with the mechanism of abnormal lipid metabolism, inflammatory response, oxidative stress and other mechanisms in modern medicine, while collateral obstruction is the internal basis and key link of the generation of toxic pathogens, which is consistent with the pathological evolution of vascular endothelial damage, plaque formation, vascular lumen stenosis and other pathologic changes in modern medicine (Ning et al., 2019).

At present, the clinical treatment pays attention to the application of “detoxification and dredging collaterals” method, flexibly adopts the methods of clearing heat and detoxification, promoting blood circulation and removing blood stasis, searching wind and relieving spasm, expelling blood stasis and dredging collaterals. Therefore, the TCM treatment theory is to combine herbs that have the effects of detoxification (clearance as a pathogenic factor or pathological product), promoting blood circulation (anticoagulation), and dredging collaterals (which can be understood as dredging blood vessels). Combined with the theory of TCM, the use of drugs needs to be based on the characteristics of patients, so a class of drugs that with the term “Detoxification and dredging collaterals” provides a new idea and scheme for TCM treatment of CAS.

AS is the fundamental driver of the pathogenesis of coronary artery disease and ischemic stroke, influenced by complex interactions among factors such as environment, genetics, susceptible genetic lesions, lipid metabolism disorders and somatic cell mutations (Bian and Jin, 2016), high CD147 expression (Liu L. et al., 2021), and serum amyloid A (Chen et al., 2021). These interactions ultimately culminate in the development of chronic inflammation, leading to CAS (Li et al., 2017). In TCM, the pathogenesis of CAS, referred to as “toxic damage and collateral resistance,” closely aligns with the inflammatory response, lipid metabolism disorder, endothelial injury, and angiogenesis seen in CAS pathogenesis in western medicine (Ning et al., 2019). Some scholars have proposed that TCM’s theory of “Arteries and veins producing phlegm nucleus” is related to the pathogenesis of CAS, suggesting that the visible “phlegm nucleus” is the stable plaque in atherosclerosis (Wenquan et al., 2019). Meanwhile, others have proposed that CAS aligns with the concept of “blood coagulation without flow” in pulse paralysis (Sha et al., 2020). Additionally, pathological neovascularization in AS plaques accelerates the development of CAS lesions, increasing plaque vulnerability (Ross et al., 2001).

In contrast, the application of detoxification and dredging collaterals for treatment of CAS can significantly improve inflammatory factors, vascular endothelial injury, blood lipids, related ultrasound plaque parameters, and other indicators, as shown in our systematic review. Therefore, further research of herbal formulas based on detoxification and dredging collaterals is crucial for enhancing the efficacy and safety of botanical drug for CAS. To the best of our knowledge, our study represents the first systematic review to investigate the efficacy and safety of detoxification and dredging collaterals herbal formulas for CAS, marking a significant step forward in the treatment of the disease.

### 4.2 Heterogeneity and variation across studies

Clinical heterogeneity was determined based on the characteristics of the participants, duration of the studies, duration of the remedy treatment, and other parameters that affect the studies. Heterogeneity among the RCTs included factors such as number of patients, duration of the treatment, and treatments of control groups. The duration of treatment and treatment of the control group were heterogeneous among the RCTs, and sensitivity analysis was conducted to eliminate heterogeneity. *I* ^2^, representing the statistical heterogeneity, was 0% in OR measurements. This indicated no heterogeneity in this analysis. However, the mean difference *I* ^2^ = 82% among the four RCTs suggests high heterogeneity, which may be related to the disease status of the patients and the long-term medication required for managing CAS as a chronic condition. Simultaneously, the administration of herbal formulations has consistently been tailored to individual patient circumstances, lacking a uniform and standardized prescription regarding the duration of TCM usage. In the future, the duration of the remedy treatment of CAS should be unified to determine the efficacy of the remedies to improve TCM on CAS for future clinical usage.

### 4.3 Strengths and limitations

Compared with the previous four meta-analyses (Hu et al., 2018; Li et al., 2019; Li et al., 2020; Zhao and Fang, 2022), this study is innovative in its use of detoxification and collateral dredging TCM as the main intervention measures. Instead of focusing on one medicine or one herbal formula, we focused on one intervention for TCM syndromes, namely, the regulation and dredging collaterals, which especially embody TCM features. In addition, we performed nearly comprehensive assessments of multiple clinical outcome measures to evaluate treatment efficacy. However, this study has some limitations. First, most RCTs included in this study did not mention the concealment of randomized assignment schemes that will lead to the influence of selective bias and measurement bias of outcome evaluation on the results of this study cannot be excluded. Second, we did not have access to patient-level data, which also limited the analysis we were able to perform. Third, we were unable to perform meta-regression to test for the primary outcome associated with different individual confounders, and each study contained a small number of patients, potentially leading to substantial heterogeneity. Lastly, the lack of universal case inclusion criteria or diagnostic tests could lead to a misdiagnosis.

At present, TCM is widely used in the treatment of CAS in China; however, the methodological quality score is generally low. Future studies are warranted to describe in detail the randomized methods used, apply appropriate randomized concealment, and try to use blinded methods. Withdrawal or loss of follow-up of cases should be recorded on time. Efficacy indicators should align with clinical guidelines, and reporting of adverse reactions should be comprehensive to enhance study quality. Future studies are expected to explore pharmacodynamic material basis and chemical structures of TCM for CAS by means of both basic and clinical research.

## 5 Conclusion

Dredging collaterals formula had curative effects and safety in CAS treatment, especially in improving carotid IMT, carotid atherosclerotic PA, carotid atherosclerotic plaque course integral, and blood lipid. This meta-analysis provides a systematic review of the treatment of CAS using TCM by elaborating on the correlation between the method of removing toxic materials and dredging collaterals and CAS mechanisms, with the aim of providing new CAS prevention and treatment strategies. In the future, large-sample, multi-center, more rigorous, and high-quality clinical trials are still needed to develop more optimized TCM treatment for CAS to benefit more patients.

## Data Availability

The original contributions presented in the study are included in the article/Supplementary Material, further inquiries can be directed to the corresponding authors.
