# Supplementary material for: Herbal formulas for detoxification and dredging collaterals in treating carotid atherosclerosis: a systematic review and meta-analysis
Source: Front Pharmacol. 2023 Dec 11;14:1147964. doi: 10.3389/fphar.2023.1147964 (PMC10749340; doi:10.3389/fphar.2023.1147964)
Supplement: Supplementary file 1 [file DataSheet1.pdf]

Appendix 1. Standard formulation of Herbal formulas for Detoxification and Dredging collaterals

Herbal formulas 01: Neixiao Ruanmai Tang granules - Wenquan Su 2021[15]

ID	Complete species name
1	*Bombyx mori* Linnaeus [Sericidae; Bombyx Batryticatus]
2	*Angelica dahurica* (Fisch. ex Hoffm.) Benth. et Hook. F. & *Angelica dahurica* (Fisch. ex Hoffm.) Benth. et Hook. f. var. *formosana* (Boiss.) Shan et Yuan [Apiaceae; Angelicae Dahuricae Radix]
3	*Mentha haplocalyx* Briq. [Lamiaceae; Menthae Haplocalycis Herba]
4	*Sinapis alba* L. & *Brassica juncea* (L.) Czern. et Coss. [Brassicaceae; Sinapis Semen]
5	*Citrus reticulata* Blanco [Rutaceae; Citri Reticulatae Pericarpium]
6	*Trionyx sinensis* Wiegmann [Trionychidae; Trionycis Carapax]
7	Commiphora myrrha (T. Nees) Engl. [Burseraceae; Myrrha]
8	*Boswellia carterii* Birdw. & *Boswellia hhaw-dajiana* Birdw. [Burseraceae; Olibanum]
9	*Salvia miltiorrhiza* Bunge [Lamiaceae; Salviae Miltiorrhizae Radix et Rhizoma]
10	*Angelica sinensis* (Oliv.) Diels [Apiaceae; Angelicae Sinensis Radix]
11	*Poria cocos* (Schw.) Wolf [Polyporaceae; Poria]
12	*Sargassum pallidum* (Turn.) C. Ag. [Sargassaceae; Sargassum]
13	*Coptis chinensis* Franch. [Ranunculaceae; Coptidis Rhizoma]
14	*Pinellia ternata* (Thunb.) Breit. [Araceae; Pinelliae Rhizoma]
15	*Curcuma Longa* L. [Zingiberaceae; Curcumae Longae Rhizoma]
16	*Lonicera japonica* Thunb. [Caprifoliaceae; Lonicerae Japonicae Flos]
17	*Citrus reticulata* Blanco [Rutaceae; Citri Reticulatae Semen]
18	*Laminaria japonica* Aresch. [Laminariaceae; Laminariae Thallus Eckloniae Thallus]
19	*Ganoderma lucidum* (Leyss. ex Fr.) Karst. [Polyporaceae; Ganoderma]
20	*Manis pentadactyla* Linnaeus [Manidae; Manis Squama]
21	*Astragalus membranaceus* (Fisch.) Bge. var. *mongholicus* (Bge.) Hsiao & *Astragalus membranaceus* (Fisch.) Bge. [Fabaceae; Astragali Radix]
22	*Ostrea gigas* Thunberg & *Ostrea talienwhanensis* Crosse & *Ostrea rivularis* Gould [Ostreidae; Ostreae Concha]
23	*Trichosanthes kirilowii* Maxim. & *Trichosanthes rosthornii* Harms [Cucurbitaceae; Trichosanthis Radix]
24	*Prunella vulgaris* L. [Lamiaceae; Prunellae Spica]
25	*Cyperus rotundus* L. [Cyperaceae; Cyperi Rhizoma]
26	*Scrophularia ningpoensis* Hemsl. [Scrophulariaceae; Scrophulariae Radix]
27	*Gleditsia sinensis* Lam. [Fabaceae; Gleditsiae Spina]
28	*Fritillaria thunbergii* Miq. [Liliaceae; Fritillaria Thunbergii Bulb]

Herbal formulas 02: Bushen huatan huoxue circulation - Yao Chen 2017[16]

ID	Complete species name
1	*Citrus reticulata* Blanco [Rutaceae; Citri Reticulatae Pericarpium]
2	*Ligusticum chuanxiong* Hort. [Apiaceae; Chuanxiong Rhizoma]
3	*Salvia miltiorrhiza* Bunge [Lamiaceae; Salviae Miltiorrhizae Radix et Rhizoma]

4	*Poria cocos* (Schw.) Wolf [Polyporaceae; Poria]
5	*Lycium barbarum* L. [Solanaceae; Lycii Fructus]
6	*Nelumbo nucifera* Gaertn. [Nelumbonaceae; Nelumbinis Folium]
7	*Cistanche deserticola* Y. C. Ma [Orobanchaceae; Cistanches Herba]
8	*Panax notoginseng* (Burkill) F. H. Chen [Araliaceae; Notoginseng Radix et Rhizoma]
9	*Cornus officinalis* Siebold et Zucc. [Cornaceae; Corni Fructus]
10	*Crataegus pinnatifida* Bunge & *Crataegus pinnatifida* Bge. var. *major* N. E. Br. [Rosaceae; Crataegi Fructus]
11	*Acorus tatarinowii* Schott [Acoraceae; Acori Tatarinowii Rhizoma]
12	*Rehjnannia glutinosa* Libosch. [Scrpophulariaceae; Rehmanniae Radix]
13	*Cuscuta australis* R. Br. & *Cuscuta chinensis* Lam [Convolvulaceae; Cuscutae Semen]
14	*Epimedium brevicornu* Maxim. & *Epimedium sagittatum* (Sieb. et Zucc.) Maxim. & *Epimedium pubescens* Maxim. & *Epimedium koreanum* Naka[Berberidaceae; Epimedii Folium]

Herbal formulas 03: Sanjie Tongmai prescription - Tingting Xie 2018[17]

ID	Complete species name
1	*Citrus reticulata* Blanco [Rutaceae; Citri Reticulatae Pericarpium]
2	*Salvia miltiorrhiza* Bunge [Lamiaceae; Salviae Miltiorrhizae Radix et Rhizoma]
3	*Dalbergia odorifera* T. Chen [Fabaceae; Dalbergiae Odoriferae Lignum]
4	*Whitmania pigra* Whitman & *Hirudo nipponica* Whitman & *Whitmania acranulata* Whitman [Hirudinidae; Hirudo]
5	*Artemisia scoparia* Waldst.et Kit. & *Artemisia capillaris* Thunb. [Compositae; Artemisiae Scopariae Herba]
6	*Alisma plantago-aquatica* Linn. & *Alisma orientate* (Sam.) Juzep. [Alismataceae; Alismatis Rhizoma]
7	*Citrus aurantium* L. [Rutaceae; Aurantii Fructus]

Herbal formulas 04: Yishenhuoxue Huatan formula - Xiaoying Chen 2017[18]

ID	Complete species name
1	*Pinellia ternata* (Thunb.) Breit. [Araceae; Pinelliae Rhizoma]
2	*Citrus reticulata* Blanco [Rutaceae; Citri Reticulatae Pericarpium]
3	*Paeonia lactiflora* Pall. & *Paeonia veitchii* Lynch [Paeoniaceae; Paeoniae Radix Rubra]
4	*Paeonia suffruticosa* Andrews [Paeoniaceae; Moutan Cortex]
5	*Lycium barbarum* L. [Solanaceae; Lycii Fructus]
6	*Achyranthes bidentata* Blume [Amaranthaceae; Achyranthis Bidentatae Radix]
7	*Dioscorea opposita* Thunb. [Dioscoreaceae; Dioscoreae Rhizoma]
8	*Cornus officinalis* Siebold et Zucc. [Cornaceae; Corni Fructus]
9	*Rehmannia glutinosa* Libosch. [Scrpophulariaceae; Rehmanniae Radix]
10	*Cuscuta australis* R. Br. & *Cuscuta chinensis* Lam [Convolvulaceae; Cuscutae Semen]

Herbal formulas 05: Tongluo Jiangzhuo decoction - Zhina Cao 2021[19]

ID	Complete species name

1	*Atractylodes macrocephala* Koidz. [Asteraceae; Atractylodis Macrocephalae Rhizoma]
2	*Salvia miltiorrhiza* Bunge [Lamiaceae; Salviae Miltiorrhizae Radix et Rhizoma]
3	*Pheretima aspergillum* (E.Perrier） [Megascolecidae; Pheretima]
4	*Poria cocos* (Schw.) Wolf [Polyporaceae; Poria]
5	*Pueraria lobata* (Willd.) Ohwi [Fabaceae; Puerariae Lobatae Radix]
6	*Trichosanthes kirilowii* Maxim. & *Trichosanthes rosthornii* Harms [Cucurbitaceae; Trichosanthis Fructus]
7	*Monascus purpureus* Went. [Aspergillus; Aspergillus]
8	*Gynostemma pentaphyllum* (Thunb.) Makino [Cucurbitaceae; Gynostemma]
9	*Panax ginseng* C. A. Mey. [Araliaceae; Ginseng Radix Et Rhizoma]
10	*Panax notoginseng* (Burkill) F.H.Chen [Araliaceae; Notoginseng Radix et Rhizoma]
11	*Crataegus pinnatifida* Bunge & *Crataegus pinnatifida* Bge. var. *major* N. E. Br. [Rosaceae; Crataegi Fructus]
12	*Whitmania pigra* Whitman & *Hirudo nipponica* Whitman & *Whitmania acranulata* Whitman [Hirudinidae; Hirudo]
13	*Ginkgo biloba* L. [Ginkgoaceae; Ginkgo Folium]
14	Alisma plantago-aquatica Linn. & *Alisma orientate* (Sam.) Juzep. [Alismataceae; Alismatis Rhizoma]

Herbal formulas 06: Huatan Tongluo Decoction - Xinyan Liu 2021[20]

ID	Complete species name
1	*Bombyx mori* Linnaeus [Sericidae; Bombyx Batryticatus]
2	*Atractylodes macrocephala* Koidz. [Asteraceae; Atractylodis Macrocephalae Rhizoma]
3	*Pinellia ternata* (Thunb.) Breit. [Araceae; Pinelliae Rhizoma]
4	*Citrus reticulata* Blanco [Rutaceae; Citri Reticulatae Pericarpium]
5	*Ligusticum chuanxiong* Hort. [Apiaceae; Chuanxiong Rhizoma]
6	*Poria cocos* (Schw.) Wolf [Polyporaceae; Poria]
7	*Glycyrrhiza glabra* L. & *Glycyrrhiza uralensis* Fisch. & *Glycyrrhiza inflata* Bat. [Fabaceae; Glycyrrhizae Radix Et Rhizoma]
8	*Uncaria rhynchophylla* (Miq.) Miq. ex Havil & *Uncaria macrophylla* Wall. & *Uncaria hirsuta* Havil. & *Uncaria sinensis* (Oliv.) Havil. & *Uncaria sessilifructus* Roxb. [Rubiaceae; Uncariae Ramulus Cum Uncis]
9	*Acorus tatarinowii* Schott [Acoraceae; Acori Tatarinowii Rhizoma]
10	*Whitmania pigra* Whitman & *Hirudo nipponica* Whitman & *Whitmania acranulata* Whitman [Hirudinidae; Hirudo]
11	*Gastrodia elata* Blume [Orchidaceae; Gastrodiae Rhizoma]

Herbal formulas 07: Banxia Baizhu Tianma Decoction - Lei Kang 2020[21]

ID	Complete species name
1	*Atractylodes macrocephala* Koidz. [Asteraceae; Atractylodis Macrocephalae Rhizoma]
2	*Pinellia ternata* (Thunb.) Breit. [Araceae; Pinelliae Rhizoma]
3	*Poria cocos* (Schw.) Wolf [Polyporaceae; Poria]
4	*Citrus reticulata* Blanco [Rutaceae; Citri Exocarpium Rubrum]
5	*Gastrodia elata* Blume [Orchidaceae; Gastrodiae Rhizoma]

6	*Glycyrrhiza glabra* L. & *Glycyrrhiza uralensis* Fisch. & *Glycyrrhiza inflata* Bat. [Fabaceae; Glycyrrhizae Radix Et Rhizoma Praeparata Cum Melle]
7	*Atractylodes macrocephala* Koidz. [Asteraceae; Atractylodis Macrocephalae Rhizoma]

Herbal formulas 08: Ditan decoction add or subtract - Ruijuan Zhang 2020[22]

ID	Complete species name
1	*Atractylodes lancea* (Thunb.) DC. & *Atractylodes chinensis* (DC.) Koidz. [Asteraceae; Atractylodis Rhizoma]
2	*Citrus reticulata* Blanco [Rutaceae; Citri Reticulatae Pericarpium]
3	*Arisaema erubescens* (Wall.) Schott [Araceae; Arisaema Cum Bile]
4	*Codonopsis pilosula* (Franch.) Nannf. [Campanulaceae; Codonopsis Radix]
5	*Pheretima aspergillum* (E.Perrier） [Megascolecidae; Pheretima]
6	*Pinellia ternata* (Thunb.) Breit. [Araceae; Pinelliae Rhizoma]
7	*Poria cocos* (Schw.) Wolf [Polyporaceae; Poria]
8	*Glycyrrhiza glabra* L. & *Glycyrrhiza uralensis* Fisch. & *Glycyrrhiza inflata* Bat. [Fabaceae; Glycyrrhizae Radix Et Rhizoma]
9	*Crataegus pinnatifida* Bunge & *Crataegus pinnatifida* Bge. var. *major* N. E. Br. [Rosaceae; Crataegi Fructus]
10	*Acorus tatarinowii* Schott [Acoraceae; Acori Tatarinowii Rhizoma]
11	*Citrus aurantium* L. [Rutaceae; Aurantii Fructus Immaturus]
12	*Bambusa tuldoides* Munro [Poaceae; Bambusae Caulis in Taenias]

Herbal formulas 09: Xuefu Zhuyu decoction and Wendan Decoction add or subtract - Lin Xin 2020[23]

ID	Complete species name
1	*Bupleurum chinense* DC. & *Bupleurum scorzonerifolium* Willd. [Apiaceae; Bupleuri Radix]
2	*Eucommia ulmoides* Oliv. [Eucommiaceae; Eucommiae Cortex]
3	*Cassia obtusifolia* L. & *Cassia tora* L. [Fabaceae; Cassiae Semen]
4	*Ziziphus jujuba Mill.* var. *spinosa* (Bunge) Hu ex H. F. Chou [Rhamnaceae; Ziziphi Spinosae Semen]
5	*Citrus reticulata* Blanco [Rutaceae; Citri Reticulatae Pericarpium]
6	*Paeonia lactiflora* Pall. & *Paeonia veitchii* Lynch [Paeoniaceae; Paeoniae Radix]
7	*Ligusticum chuanxiong* Hort. [Apiaceae; Chuanxiong Rhizoma]
8	*Angelica sinensis* (Oliv.) Diels [Apiaceae; Angelicae Sinensis Radix]
9	*Pinellia ternata* (Thunb.) Breit. [Araceae; Pinelliae Rhizoma]
10	*Poria cocos* (Schw.) [Polyporaceae; Poria]
11	*Glycyrrhiza glabra* L. & *Glycyrrhiza uralensis* Fisch. & *Glycyrrhiza inflata* Bat. [Fabaceae; Glycyrrhizae Radix Et Rhizoma]
12	*Lycium barbarum* L. [Solanaceae; Lycii Fructus]
13	*Nelumbo nucifera* Gaertn. [Nelumbonaceae; Nelumbinis Folium]
14	*Carthamus tinctorius* L. [Asteraceae; Carthami Flos]
15	*Astragalus membranaceus* (Fisch.) Bge. var. *mongholicus* (Bge.) Hsiao & *Astragalus membranaceus* (Fisch.) Bge. [Fabaceae; Astragali Radix]
16	*Gynostemma pentaphyllum* (Thunb.) Makino [Cucurbitaceae; Gynostemma]

17	*Platycodon grandiflorus* (Jacq.) A. DC. [Campanulaceae; Platycodonis Radix]
18	*Achyranthes bidentata* Blume [Amaranthaceae; Achyranthis Bidentatae Radix]
19	*Crataegus pinnatifida* Bunge & *Crataegus pinnatifida* Bge. var. *major* N. E. Br. [Rosaceae; Crataegi Fructus]
20	*Prunus persica* (L.) Batsch & *Prunus davidiana* (Carr.) Franch [Rosaceae; Persicae Semen]
21	*Citrus aurantium* L. [Rutaceae; Aurantii Fructus]

Herbal formulas 10: Tongmai Jiangzhuo granules - Yingshun Bai 2019[24]

ID	Complete species name
1	*Paeonia lactiflora* Pall. [Paeoniaceae; Paeoniae Radix Alba]
2	*Bupleurum chinense* DC. & *Bupleurum scorzonerifolium* Willd. [Apiaceae; Bupleuri Radix]
3	*Ziziphus jujuba Mill.* [Rhamnaceae; Jujubae Fructus]
4	*Salvia miltiorrhiza* Bunge [Lamiaceae; Salviae Miltiorrhizae Radix et Rhizoma]
5	*Pinellia ternata* (Thunb.) Breit. [Araceae; Pinelliae Rhizoma]
6	*Scutellaria baicalensis* Georgi [Lamiaceae; Scutellariae Radix]
7	*Rheum officinale* Baill. & *Rheum palmatum* L. & *Rheum tanguticum* Maxim. ex Balf. [Polygonaceae; Rhei Radix Et Rhizoma]
8	*Panax notoginseng* (Burkill) F.H.Chen [Araliaceae; Notoginseng Radix et Rhizoma]
9	*Zingiber officinale* Roscoe [Zingiberaceae; Zingiberis Rhizoma Recens]
10	*Citrus aurantium* L. [Rutaceae; Aurantii Fructus Immaturus]

Herbal formulas 11: Detoxifying and eliminating carbuncle prescription - Rui Shi 2019[25]

ID	Complete species name
1	*Ligusticum chuanxiong* Hort. [Apiaceae; Chuanxiong Rhizoma]
2	*Salvia miltiorrhiza* Bunge [Lamiaceae; Salviae Miltiorrhizae Radix et Rhizoma]
3	*Pheretima aspergillum* (E.Perrier） [Megascolecidae; Pheretima]
4	*Nardostachys jatamansi* (D.Don) DC. [Caprifoliaceae; Nardostachyos Radix Et Rhizoma]
5	*Trichosanthes kirilowii* Maxim. & *Trichosanthes rosthornii* Harms [Cucurbitaceae; Trichosanthis Fructus]
6	*Coptis chinensis* Franch. [Ranunculaceae; Coptidis Rhizoma]
7	*Lonicera japonica* Thunb. [Caprifoliaceae; Lonicerae Japonicae Flos]
8	*Whitmania pigra* Whitman & *Hirudo nipponica* Whitman & *Whitmania acranulata* Whitman [Hirudinidae; Hirudo]
9	*Anemarrhena asphodeloides* Bunge [Asparagaceae; Anemarrhenae Rhizoma]

Herbal formulas 12: Compound Danshen dropping pills - Duanhua Cao 2019[26]

ID	Complete species name
1	*Cinnamomum camphora* (L.) J. Presl [Lauraceae; Borneolum]
2	*Salvia miltiorrhiza* Bunge [Lamiaceae; Salviae Miltiorrhizae Radix et Rhizoma]
3	*Panax notoginseng* (Burkill) F.H.Chen [Araliaceae; Notoginseng Radix et Rhizoma]

Herbal formulas 13: Huaban Tongluo Decoction - Moqun Wang 2020[27]

ID	Complete species name
1	*Pheretima aspergillum* (E.Perrier） [Megascolecidae; Pheretima]
2	*Lycium barbarum* L. [Solanaceae; Lycii Fructus]
3	*Trichosanthes kirilowii* Maxim. & *Trichosanthes rosthornii* Harms [Cucurbitaceae; Trichosanthis Fructus]
4	*Coptis chinensis* Franch. [Ranunculaceae; Coptidis Rhizoma]
5	*Astragalus membranaceus* (Fisch.) Bge. var. *mongholicus* (Bge.) Hsiao & *Astragalus membranaceus* (Fisch.) Bge. [Fabaceae; Astragali Radix]
6	*Ostrea gigas* Thunberg & *Ostrea talienwhanensis* Crosse & *Ostrea rivularis* Gould [Ostreidae; Ostreae Concha]
7	*Panax notoginseng* (Burkill) F.H.Chen [Araliaceae; Notoginseng Radix et Rhizoma]
8	*Gastrodia elata* Blume [Orchidaceae; Gastrodiae Rhizoma]

Herbal formulas 14: Dazhu Rhodiola capsule - Ying Liu 2019[28]

ID	Complete species name
1	*Rhodiola crenulata* (Hook. f. et Thoms.) H. Ohba [Crassulaceae; Rhodiolae Crenulatae Radix Et Rhizoma]
