# Supplementary material for: Herbal formulas for detoxification and dredging collaterals in treating carotid atherosclerosis: a systematic review and meta-analysis
Source: Front Pharmacol. 2023 Dec 11;14:1147964. doi: 10.3389/fphar.2023.1147964 (PMC10749340; doi:10.3389/fphar.2023.1147964)
Supplement: Supplementary file 2 [file DataSheet2.docx]

Search Strategy of Databases

The following database will be searched from inception to 1, June, 2022.

Database 1 Pubmed n=168

#1 Carotid atherosclerosis [MeSH Terms]

#2 Arter∗ Disease∗, Carotid[Title/Abstract] OR Carotid Arter∗ Disease∗[Title/Abstract] OR Artery Disorder∗, Carotid[Title/Abstract] OR Carotid Artery Disorder[Title/Abstract] OR Disorders, Carotid Artery[Title/Abstract] OR Carotid Atherosclero∗[Title/Abstract] OR Atherosclerotic Disease∗, Carotid[Title/Abstract]

#3 #1 OR #2

#4 Medicine, Chinese Traditional [MeSH Terms]

#5 Medicine, Chinese Traditional[Title/Abstract] OR Traditional Chinese Medicine[Title/Abstract] OR Traditional Medicine, Chinese[Title/Abstract] OR Zhong Yi Xue[Title/Abstract] OR Chinese Traditional Medicine[Title/Abstract] OR Chinese Medicine, Traditional[Title/Abstract] OR Jiedu[Title/Abstract] OR Tongluo[Title/Abstract] OR Zhongyao[Title/Abstract]

#6 #4 OR #5

#7 Drugs, Chinese Herbal [MeSH Terms]

#8 Drugs, Chinese Herbal[Title/Abstract] OR Chinese Drugs, Plant[Title/Abstract] OR Chinese Herbal Drugs[Title/Abstract] OR Herbal Drugs, Chinese[Title/Abstract] OR Plant Extracts, Chinese[Title/Abstract] OR Chinese Plant Extracts[Title/Abstract] OR Extracts, Chinese Plant[Title/Abstract]

#9 #6 OR #7 OR #8

#10 #9 OR #3

Database 2 Web of science n=38

#1 Carotid atherosclerosis (主题) or Arter∗ Disease∗, Carotid (主题) or Carotid Arter∗ Disease∗ (主题) or Artery Disorder∗, Carotid (主题) or Carotid Artery Disorder (主题) or Carotid Atherosclero∗ (主题) or Disorders, Carotid Artery (主题) or Atherosclerotic Disease∗, Carotid (主题)

#2 Medicine, Chinese Traditional (主题) or Traditional Chinese Medicine (主题) or Traditional Medicine, Chinese (主题) or Zhong Yi Xue[Title/Abstract] (主题) or Chinese Traditional Medicine (主题) or Chinese Medicine, Traditional (主题) or Jiedu (主题) or Tongluo (主题) or Zhongyao (主题)

#3 Drugs, Chinese Herbal (主题) or Chinese Drugs, Plant (主题) or Chinese Herbal Drugs (主题) or Herbal Drugs, Chinese (主题) or Plant Extracts, Chinese (主题) or Chinese Plant Extracts (主题) or Extracts, Chinese Plant (主题)

#4 #2 OR #3

#5 #1 AND #4

Database 3 Medline n=107

#1 Carotid atherosclerosis (主题) or Arter∗ Disease∗, Carotid (主题) or Carotid Arter∗ Disease∗ (主题) or Artery Disorder∗, Carotid (主题) or Carotid Artery Disorder (主题) or Carotid Atherosclero∗ (主题) or Disorders, Carotid Artery (主题) or Atherosclerotic Disease∗, Carotid (主题)

#2 Medicine, Chinese Traditional (主题) or Traditional Chinese Medicine (主题) or Traditional Medicine, Chinese (主题) or Zhong Yi Xue(主题) or Chinese Traditional Medicine (主题) or Chinese Medicine, Traditional (主题) or Jiedu (主题) or Tongluo (主题) or Zhongyao (主题)

#3 Drugs, Chinese Herbal (主题) or Chinese Drugs, Plant (主题) or Chinese Herbal Drugs (主题) or Herbal Drugs, Chinese (主题) or Plant Extracts, Chinese (主题) or Chinese Plant Extracts (主题) or Extracts, Chinese Plant (主题)

#4 #2 OR #3

#5 #1 AND #4

Database 4 Embase (Ovid)=9

#1 'carotid atherosclerosis':ti,ab,kw OR 'arter∗ disease∗, carotid':ti,ab,kw OR 'carotid arter∗ disease∗':ti,ab,kw OR 'artery disorder∗, carotid':ti,ab,kw OR 'carotid artery disorder':ti,ab,kw OR 'carotid atherosclero∗':ti,ab,kw OR 'disorders, carotid artery':ti,ab,kw OR 'atherosclerotic disease∗, carotid':ti,ab,kw

#2 'medicine, chinese traditional':ti,ab,kw OR 'traditional chinese medicine':ti,ab,kw OR 'traditional medicine, chinese':ti,ab,kw OR 'zhong yi xue':ti,ab,kw OR 'chinese traditional medicine':ti,ab,kw OR 'chinese medicine, traditional':ti,ab,kw OR jiedu:ti,ab,kw OR tongluo:ti,ab,kw OR zhongyao:ti,ab,kw

#3 'drugs, chinese herbal':ti,ab,kw OR 'herbaceous agent':ti,ab,kw OR 'chinese drugs, plant':ti,ab,kw OR 'chinese herbal drugs':ti,ab,kw OR 'herbal drugs, chinese':ti,ab,kw OR 'plant extracts, chinese':ti,ab,kw OR 'chinese plant extracts':ti,ab,kw OR 'extracts, chinese plant':ti,ab,kw

#4 #2 OR #3

#5 #1 AND #4

Database 5 CNKI (China National Knowledge Infrastructure) n=938

(TKA='解毒' OR TKA='通络' OR TKA='中药' OR TKA='中医') AND (TKA='动脉狭窄' OR TKA='动脉斑块' OR TKA='动脉粥样硬化' OR TKA='动脉硬化' OR TKA='脂质代谢障碍') AND (TKA='颈' OR TKA='颈部' OR TKA='头颈') AND (TKA='临床研究' OR TKA='临床实验' OR TKA='随机' OR TKA='盲法' OR TKA='双盲')

Database 6 WanFang (WanFang data) n=232

(题名或关键词=(解毒 OR 通络 OR 中药 OR 中医)) AND (题名或关键词=(动脉狭窄 OR 动脉斑块 OR 动脉粥样硬化 OR 动脉硬化 OR 脂质代谢障碍)) AND (题名或关键词=(颈 OR 颈部 OR 头颈)) AND (题名或关键词=(临床研究 OR 临床实验 OR 随机 OR 盲法 OR 双盲))

Database 7 VIP (VIP Database for Chinese Technical Periodicals) n=58

(M=(解毒 OR 通络 OR 中药 OR 中医)) AND (M=(动脉狭窄 OR 动脉斑块 OR 动脉粥样硬化 OR 动脉硬化 OR 脂质代谢障碍)) AND (M=(颈 OR 颈部 OR 头颈)) AND (M=(临床研究 OR 临床实验 OR 随机 OR 盲法 OR 双盲))

Database 8 Sinomed n=1110

#1 "解毒"[摘要:智能] OR "通络"[摘要:智能] OR "中药"[摘要:智能] OR "中医"[摘要:智能]

#2 "动脉狭窄"[摘要:智能] OR "动脉粥样硬化"[摘要:智能] OR "动脉斑块"[摘要:智能] OR "动脉硬化"[摘要:智能] OR "脂质代谢障碍"[摘要:智能]

#3 "颈"[摘要:智能] OR "颈部"[摘要:智能] OR "头颈"[摘要:智能]

#4 (#3) AND (#2) AND (#1)
